# Antineutrophil Cytoplasmic Antibody-Negative Pauci-Immune Crescentic Glomerulonephritis in Systemic Lupus Erythematosus: A Case Report and Literature Review

**DOI:** 10.7759/cureus.87285

**Published:** 2025-07-04

**Authors:** Mohammed A Miqdad, Zainulabdeen S Al-Saedi, Lina Alatta, Hasan Hulwi, Sheng Kuo

**Affiliations:** 1 Research, Michigan State University, East Lansing, USA; 2 Nephrology, NewYork-Presbyterian, New York, USA; 3 Medicine/Nephrology, NewYork-Presbyterian Queens, New York, USA; 4 Nephrology, NewYork-Presbyterian Queens, New York, USA

**Keywords:** anca-negative, glomerulonephritis (gn), lupus nephritis, pauci-immune glomerulonephritis (gn), rituximab (rtx)

## Abstract

Lupus nephritis (LN) is a common complication of systemic lupus erythematosus (SLE) and often presents as an immune complex-mediated glomerular disease. While pauci-immune crescentic glomerulonephritis (PICG) is commonly associated with antineutrophil cytoplasmic antibodies (ANCAs), its occurrence in the context of LN is rare. We report a case of an SLE patient who developed acute kidney injury due to ANCA-negative PICG. The kidney injury was severe enough to necessitate renal replacement therapy; however, the patient responded well to treatment. We also review the literature and previously reported cases of PICG in patients with SLE.

## Introduction

Pauci-immune crescentic glomerulonephritis (PICG) is characterized by minimal or absent glomerular immunoglobulin deposition on immunofluorescence or electron microscopy and is marked histologically by necrotizing and crescentic glomerulonephritis [1,2]. It is among the most common causes of rapidly progressive glomerulonephritis. PICG is most often associated with antineutrophil cytoplasmic antibody (ANCA)-associated vasculitis; however, a subset of patients with PICG are ANCA-negative [1,2]. Reported incidence rates range from 2% to 6% in general, but may reach as high as 33-49.5% in Asian populations. PICG has also been documented in association with several autoimmune diseases, including systemic lupus erythematosus (SLE) [2].

In SLE, the most common renal manifestation is prototypical immune complex-mediated glomerulonephritis. PICG, analogous to ANCA-associated renal vasculitis, is rarely seen in lupus nephritis (LN) and is not included in the International Society of Nephrology/Renal Pathology Society (ISN/RPS) classification system. An overlap syndrome of LN and ANCA-associated vasculitis is rare, with a reported prevalence of approximately 2% [3].

We present a case of a patient with systemic lupus who developed severe acute kidney injury due to ANCA-negative PICG and responded well to immunosuppressive therapy. We also reviewed the literature and previously reported cases of PICG in patients with SLE.

## Case presentation

A 30-year-old female was diagnosed with SLE at the age of 20 based on clinical findings of arthritis and positive serologies, including antinuclear antibody (ANA), double-stranded DNA antibody (dsDNA), and anti-Ro/SSA. She had regular rheumatology follow-ups but used hydroxychloroquine inconsistently. Routine laboratory testing performed four months prior to presentation revealed a WBC count of 2.5 × 10³/µL, neutrophils at 1.02 × 10³/µL, hemoglobin at 13.0 g/dL, platelets at 201 × 10³/µL, serum creatinine at 0.78 mg/dL, negative urinalysis for blood and protein, and a spot urine protein-to-creatinine ratio (PCR) of 0.07 g/g of creatinine.

She presented to the ED with a three-day history of epigastric pain, nausea, poor appetite, nonbloody vomiting, chills, and sweating. She also reported decreased urine output starting two days prior to admission. There were no associated symptoms of shortness of breath, skin rash, joint pain, alopecia, oral ulcers, chest pain, or Raynaud’s phenomenon. She denied any recent changes in medication and had not used nonsteroidal anti-inflammatory drugs.

Initial vital signs were within normal limits, and physical examination was unremarkable. Laboratory evaluation was notable for a WBC count of 20.53 × 10³/µL with neutrophilic predominance, Hgb of 12 g/dL, platelets 148 × 10³/µL, no peripheral eosinophilia or schistocytes, blood urea nitrogen 41.4 mg/dL, and serum creatinine 6.57 mg/dL. Urinalysis was positive for bacteria, showed 5 RBCs and 2 WBCs per high-power field, protein >1000 mg/dL, small blood, and was negative for nitrites and leukocyte esterase. Urine PCR could not be calculated due to a urine protein concentration >600 mg/dL and urine creatinine of 465.9 mg/dL.

The patient’s kidney function continued to deteriorate, as detailed in Table 1. Because she was admitted over the weekend, a kidney biopsy was not performed until hospital day 3. She began hemodialysis due to clinical signs of uremia. Given a high clinical suspicion for LN, pulse methylprednisolone at 1 g daily for three days was initiated. Ceftriaxone was administered in the ED for leukocytosis and urinalysis findings and was continued for seven days. Unfortunately, a urine culture was not obtained before starting antibiotics, and the culture collected after antibiotic initiation was negative.

**Table 1:** Basic metabolic panel results during hospital admission

Parameter	Day 1	Day 2	Day 3	Day 5	Day 7	Day 9	Day 10	Day 11	Day 12	Reference range
Sodium, mmol/L	133	133	131	133	135	137	137	142	144	136-145
Potassium, mmol/L	3.4	3.9	3.6	4.2	3.7	3.7	4.1	4.2	3.9	3.5-5.1
Chloride, mmol/L	95	97	96	95	98	100	100	105	107	98-107
Bicarbonate, mmol/L	19	19	17	17	22	22	23	25	24	22-29
Blood urea nitrogen, mg/dL	41.4	50.2	66.7	93.4	81.1	64.6	53	43.2	31.4	8-23
Creatinine, mg/dL	6.57	8.08	10.84	11.15	6.37	2.58	2.01	1.74	1.46	0.50-0.95
Calcium, mg/dL	8.8	8.5	9	9.4	8.6	8.8	8.7	8.8	8.8	8.6-10.4
Anion gap	19	17	18	21	15	15	14	12	13	5-17

Subsequent serological and immunological testing revealed an ANA titer of 1:2560, dsDNA titer of 1:1280, SSA (anti-Ro) of 128 AU/mL, and rheumatoid factor of 21.9 IU/mL. SSB, anti-PR3, anti-MPO (via ELISA), C3, and C4 were within normal limits. The patient received a total of three hemodialysis sessions, with evidence of renal recovery by hospital day 7 - two days after completing the pulse steroid course.

Renal pathology demonstrated active crescentic glomerulonephritis of the pauci-immune type, with no evidence of glomerular immune complex deposition. The patient received 1 g of rituximab along with a steroid taper and was discharged home without the need for continued renal replacement therapy. She was scheduled for follow-up with rheumatology for the next dose of rituximab and with nephrology for continued management of PICG.

**Figure 1:**
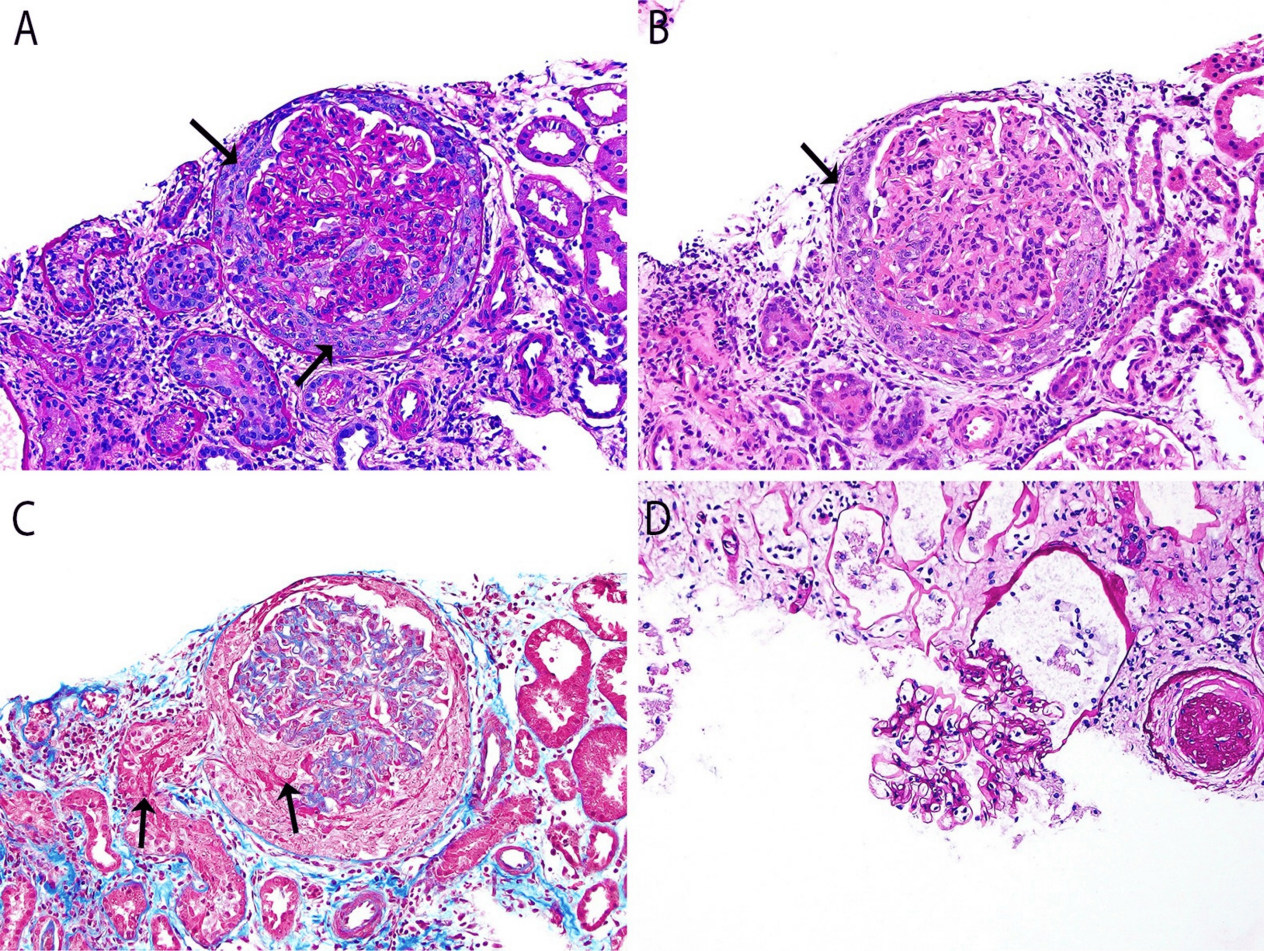
Light microscopy Global, nearly sequential necrotizing and cellular crescent formation.

The biopsy included a total of 21 glomeruli, one of which was globally sclerosed (1/21). One glomerulus demonstrated a global, nearly sequential necrotizing and cellular crescent (Figure 1). The remaining glomeruli appeared unremarkable, with no evidence of mesangial or endocapillary proliferation. Widespread acute tubular injury with focal necrosis was also observed.

**Figure 2:**
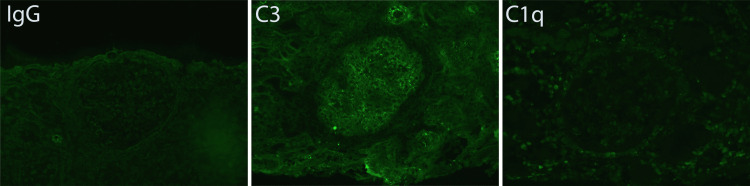
Immunofluorescence microscopy Glomerular staining shows a granular pattern along the capillary walls for trace (+) C3, interpreted as nonspecific binding.

Immunofluorescence analysis was performed on six glomeruli (Figure 2). Staining revealed a granular pattern along the glomerular capillary walls for trace (+) C3, which was considered nonspecific. The glomeruli were negative for IgG, IgA, IgM, C1q, and both kappa and lambda light chains. The tubulointerstitial compartment also showed no staining for immunoglobulins, complement components, or light chains.

**Figure 3:**
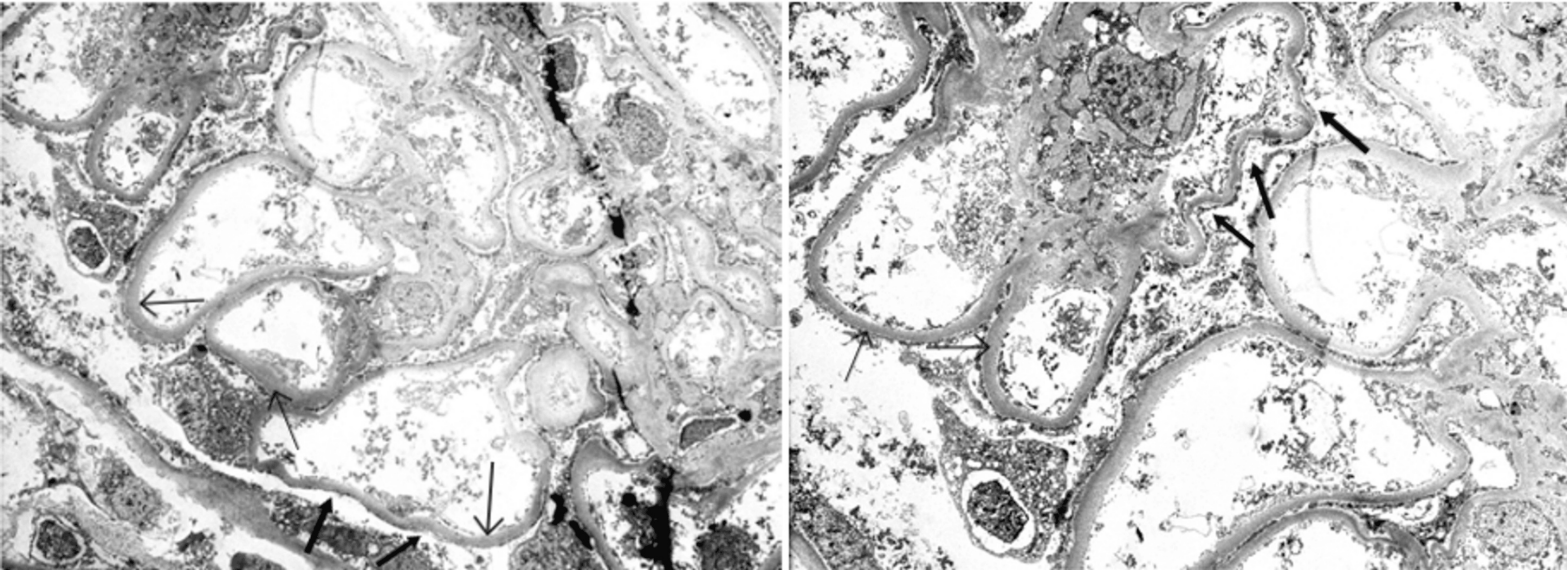
Electron microscopy The glomerular capillary basement membrane exhibited a smooth and regular external contour and texture but was moderately thickened (thin arrow). Significant foot process effacement was observed, involving approximately 50% of the basement membrane surface (thick arrow).

Electron microscopy of one glomerulus showed that the glomerular capillary basement membrane had a smooth and regular external contour and texture but was moderately thickened, measuring between 650 and 900 nm (mean 750 nm; thin arrow; Figure 3). Significant foot process effacement was observed, involving approximately 50% of the basement membrane surface (thick arrow). The mesangial areas were unremarkable, and no immune complex-type electron-dense deposits were identified.

## Discussion

PICG is one of the most frequent causes of rapidly progressive glomerulonephritis. It is characterized by glomerular necrotizing crescents with absent or minimal immunoglobulin staining on immunofluorescence and lack of immune deposits on electron microscopy [4,5]. While ANCAs are present in approximately 80-90% of PICG cases, ANCA-negative cases have also been reported. In one of the largest retrospective analyses comparing 28 patients with ANCA-negative PICG to those with ANCA-positive disease, the ANCA-negative group exhibited more proteinuria, a higher prevalence of nephrotic syndrome, and more extensive and fulminant glomerular injury, findings associated with poorer renal outcomes, yet had fewer extra-renal manifestations [4].

ANCA-negative PICG may be triggered by bacterial infections, including *Klebsiella pneumoniae*, *Streptococcus parasanguinis*, and *Staphylococcus epidermidis*, with renal involvement typically occurring within zero to three days of infection and presenting with or without low serum C3 levels. It is important to differentiate bacterial-associated PICG from infection-associated glomerulonephritis, as the latter is histologically defined by endocapillary proliferation and prominent C3 deposition. Additionally, ANCA-negative PICG has been associated with progressive malignancies such as marginal zone lymphoma and lung adenocarcinoma. Notably, in some cases, the diagnosis of ANCA-negative PICG preceded evidence of malignancy progression, even when the malignancy was believed to be in remission. Certain medications have also been implicated in the development of ANCA-negative PICG, including anti-vascular endothelial growth factor therapy used in uterine adenocarcinoma and hormonal treatments for in vitro fertilization. Ronsin et al. classified ANCA-negative PICG as either primary, in which no coexisting disease is identified, or secondary, due to infection, malignancy, and, less commonly, medication exposure [5].

Although rare, ANCA-negative PICG has been reported in patients with SLE, sometimes referred to as “pauci-immune lupus nephritis.” In these cases, features of lupus activity may still be present, such as a positive Coombs test and low serum C3 levels, despite the absence of C3 deposition [6]. For example, Li et al. described a case of pauci-immune LN in a patient with hemolytic anemia, hypoalbuminemia, nephrotic-range proteinuria, and low C3 and C4 levels, alongside histologic findings consistent with PICG [7]. Similarly, Liebowitz et al. reported an SLE case with PICG characterized by low serum C3 and weak glomerular basement membrane deposits of IgG, IgM, and kappa, but no C3 deposition; a repeat kidney biopsy later revealed class II LN [8]. Beyond SLE, PICG has also been observed in other connective tissue diseases [9] and in Fabry disease [10].

Treatment strategies for PICG are variable across reports. In one SLE case, the initial kidney biopsy revealed PICG with two fibrocellular crescents and negative immunofluorescence staining. Although serum creatinine was within normal range, the patient was treated with methylprednisolone followed by oral prednisone and a six-month course of cyclophosphamide, resulting in complete resolution of proteinuria and microscopic hematuria and normalization of serum albumin and complement levels [11]. Similar treatment regimens combining corticosteroids and cyclophosphamide have been used successfully in other cases [6,11-13]. Rituximab, corticosteroids, and mycophenolate were used in the case reported by Liebowitz et al. [8].

Based on our review of the literature, there appears to be no consistent clinical presentation of PICG in patients with SLE. Given the limited available data, primarily case reports, much remains unknown about its etiology and pathophysiology. The absence of immune deposits suggests that ANCA-negative PICG may result from antibody-independent, cell-mediated immune injury, potentially triggered by infection (as suspected in our case), malignancy, or chemical agents. Thus, a diagnosis of ANCA-negative PICG should prompt a comprehensive evaluation for possible infectious, malignant, or drug-related causes. While optimal treatment is not well defined, immunosuppressive therapy remains essential. Encouragingly, our patient responded well to a combination of antibiotics and immunosuppressive treatment. It would be of interest to determine whether a follow-up kidney biopsy might reveal immune complex lesions, suggesting that PICG could represent a spectrum of LN.

## Conclusions

ANCA-negative PICG is an exceptionally rare presentation in SLE. Its precise pathogenesis, optimal management, and prognosis remain unclear, with most of the current evidence derived from isolated case reports and case series. Although there is no established consensus on treatment, current evidence supports the use of corticosteroids in combination with cyclophosphamide or rituximab. Importantly, all potential causes of ANCA-negative PICG, particularly infection and malignancy, should be thoroughly excluded. We described a case of SLE with acute kidney injury and PICG, who showed a favorable response to immunosuppressive therapy. Moving forward, we hope that this rare entity may be recognized and incorporated into clinical guidelines for LN.
